# Integration of human papillomavirus associated anal cancer screening into HIV care and treatment program in Pakistan: perceptions of policymakers, managers, and care providers

**DOI:** 10.1186/s12889-023-15896-1

**Published:** 2023-05-31

**Authors:** Muslima Ejaz, Anna Mia Ekström, Tazeen Saeed Ali, Mariano Salazar, Alyan Ahmed, Dania Ali, Ayman Haroon, Sameen Siddiqi

**Affiliations:** 1 Department of Global Public Health, Karolinska Institutet Stockholm, Widerströmska Huset 18 A 171 77, Stockholm, Sweden; 2 Department of Community Health Sciences, Aga Khan University, Stadium Road, P.O. Box 3500, Karachi, 74800 Pakistan; 3 Department of Infectious Diseases, South Central Hospital, Stockholm, Sweden; 4 School of Nursing, Aga Khan University, Karachi, Pakistan; 5 Department of Biostatistics and Epidemiology, School of Public Health, Boston University, Boston, MA USA

**Keywords:** HPV, HIV, MSM, Transgender women, Anal cancer screening, Integration, Pakistan

## Abstract

**Background:**

The incidence of anal cancer, largely associated with anal human papillomavirus (HPV) infection, is increasing among men who have sex with men (MSM), and transgender women living with or without HIV. Screening for anal cancer to detect anal precancerous lesions in high-risk groups is an important opportunity for prevention but still lacking in many low-and-middle-income countries. The aim of this study was to explore the readiness of Pakistan’s healthcare system to integrate anal cancer and HPV screening into a national HIV program, as perceived by policymakers, health managers, and healthcare providers.

**Design:**

This qualitative study using key-informant interviews with participants influence in policy making, implementation and advocacy from public and private sector were conducted between March 2021 to August 2021 in Karachi Pakistan.

**Methods:**

Key informants were purposely selected from different domains of the healthcare system responsible for the target group of interest, MSM and transgender-women in general and people living with HIV in particular. A total of 18 key informants, at different levels of seniority were recruited from governmental and non-governmental organizations, high-level infectious disease healthcare managers, and United Nations Program representatives. Qualitative content analysis was used to identify the manifest and latent themes, based on socioecological framework.

**Results:**

The results were grouped into five major themes; (1) The policy context and priorities, (2) Health systems factors, (3) Community environment, (4) Healthcare setting & providers and (5) Individual-level obstacles. The policy actors expressed their concerns about their limited voice in country’s health and health related priority setting. Informants reported a lack of political will and suggested that government should bring a change in the paradigm of healthcare service delivery from reactive to proactive approach. Although, participants unanimously favored integration of HPV preventive services into existing HIV program, they also identified several service delivery barriers including trained workforce shortage, limited capacity of information technology, lack of supplies needed for screening, lack of financing, and lack of services that could meet key-populations needs. Participants also predicted other implementation challenges such as stigma, social victimization, and systemic discrimination against at-risk groups at healthcare facilities.

**Conclusion:**

Although policy makers and health providers in Pakistan saw a clear need to scale-up and integrate anal cancer screening for key populations, the feasibility of this is dependent on political will, financing, anti-stigma and discrimination interventions and health system efficiency.

## Background

Human papillomaviruses (HPV) are the most common sexually transmitted viral infections worldwide [1]. High-risk HPV types - predominantly 16 and 18 are involved in almost 100% of cervical cancer [2]. Persistent infection with high-risk HPV16 is associated with 88% of anal cancer [3] and with some head and neck cancers [4]. Certain at-risk groups such as gay, bisexual, and other men who have sex with men (MSM) particularly those MSM who are infected with HIV are at increased risk of HPV infection and are disproportionately affected by HPV associated genital warts, anal cancer and its precursor high-grade squamous intraepithelial lesions [5]. Recently study conducted in Pakistan by our group found 65% prevalence of HPV infection [6] and 35% of anal precancerous lesions [7] in MSM and transgender women living with and without HIV. HPV infection is an important risk factor for both cervical and anal cancers and causes similar type of squamous intraepithelial precancerous lesions, cervical intraepithelial neoplasia (CIN) and anal intraepithelial neoplasia (AIN), respectively. There is a known sequence from persistent HPV infection to low- to high-grade squamous intraepithelial lesions (HSIL), and finally, to invasive cancer [8].

A screening process similar to the one for the cervix has been described for the anus, using anal cytology as the first screening approach, with referral of those with abnormalities to high-resolution anoscopy (HRA) [9]. A recent meta-analysis showed that anal cytology has a sensitivity and specificity similar to cervical Pap screening (90% and 33%, respectively) [10]. Anal cytological screening and treatment of dysplastic lesions have been proposed as a way of reducing the burden of anal cancer [11], but there are no national or international guidelines for at-risk population [12]. However, several research studies and organizations [13, 14] have described anal cytology as a possible screening method for MSM [9] and in particular for men, women and MSM, living with HIV [15, 16, 17]. As early detection of anal cancer precursors improves survival many clinics in different parts of the world have implemented screening program for anal cancer [18–21]. A recent randomized trial has determined the efficacy of treating anal precancerous lesions i.e., HSIL and had showed efficacy in reducing the incidence of anal cancer, underscoring support for the use of screening and treatment for anal HSIL as the standard of care for persons living with HIV who are 35 years of age or older also for all populations at increased risk of anal cancer [22, 23]. Moreover, recently (2023) RP van der Zee et al. have examined trends in incidence of and mortality after anal cancer diagnosis in MSM living with HIV, including the effect of screening from 2007 onwards in the Netherlands. The authors have reported the decline in the anal cancer rates as well as decrease in the crude anal cancer associated 5-year mortality in MSM living with HIV [24]. Furthermore the authors have concluded that because of being diagnosed at an early disease stage they had better survival, which is an important justification to screen those most at risk of anal cancer [24]. The primary preventive measure to reduce HPV related disease is vaccination. Data from HPV vaccine trials supports the benefits of HPV vaccination among at-risk population such as women [25] and men [26] living with HIV, as well as both HIV negative and positive MSM [27].

The dual epidemics of HIV and HPV affect similar at-risk populations. In Pakistan the prevalence of HIV is over 9% in MSM and 13% in transgender women [28]. The Pakistani government is running a national program for HIV care and treatment, and also provide comprehensive HIV testing and awareness services through community-based organizations (CBOs) which are often run by key populations like MSM, transgender women, or female sex workers. However, despite the high prevalence of HPV infection [6] and anal precancerous lesions [7] in these key populations neither the government nor any CBO currently offer any kind of organized services for HPV prevention. This, indeed, is a missed opportunity for prevention, given that the HPV vaccine and Pap tests have been effective cancer prevention tools [29]. Moreover, while infrastructure for treating detected invasive anal cancer remains a concern in low- and middle-income countries (LMICs), effective early detection and treatment of pre-invasive disease may be of considerable importance.

With the study aim to assess the policy context at national and lower levels, and to evaluate the health systems readiness for the introduction of HPV screening and HPV vaccination for high-risk key populations in Pakistan, we explored the perceptions of policy makers, health administrators, health care providers and the program managers of CBOs working for this sexual and gender minorities.

## Methods

### Study design

To explore the systems and policy readiness to provide HPV associated including anal cancer screening services as well as HPV vaccination, we used a qualitative exploratory research design [30], including key informants interviews with senior policy makers and programmers.

### Study setting

In Pakistan, the healthcare delivery system consists of public and a comparatively more dominant private sector. In general, the responsibility for provision of healthcare is province based, however, studies have shown that Pakistan’s private sector healthcare system is outperforming the public sector healthcare system in terms of service quality and patient satisfaction, with 70% of the population being served by the private health sector [31]. The Ministry of National Health Services, Regulation and Coordination is predominantly responsible for policy making, coordination, technical support and seeking foreign assistance (funding and technical support) at the federal level. While the provincial and district departments of health are responsible for the delivery and management of health services. Health service delivery is organized through preventive, promotive, curative, and rehabilitative services. Curative and rehabilitative services are being provided mainly at secondary and tertiary care facilities. Preventive and promotive services are offered through various national programs, basic and rural health care centers, and community health workers’ interfacing with the communities through primary healthcare facilities and outreach activities.

The organizational structure at the provincial level includes various wings such as, technical, administrative, planning, development, and procurement, monitoring and inspection.

It is important to note that for sexual and gender minorities including MSM and transgender women, health care services are provided through the National AIDS Control Program under the Ministry of Health. This program provides comprehensive voluntary counseling and laboratory testing for HIV and other sexually transmitted infections (STIs), (but HPV not yet included as an STI) and antiretroviral therapy for HIV through a community-based organization model.

### Participants and sampling procedure

A maximum variation sampling [32] was employed to select key informants with influence in policy making, implementation and advocacy. The participants were recruited among stakeholders including policy makers (n = 3), program managers (n = 5) implementation staff (n = 5) and senior clinicians representing both public, private and CBOs (n = 5). Potential key-informants were identified through professional networks of the first and the last author and an introductory email was sent outlining the study and its objectives. Interested informants were then sent an information sheet and a time was arranged for interview. Then participants were asked to identify another person who might be suitable for the interview using snowball sampling technique.

### Data collection

Twenty-four potential informants were contacted; of whom, 18 participants agreed to be interviewed. Recruitment of participants ended when further sampling would not generate any new concepts or information, defined as the point of saturation [33]. The informants were from international UN program and from Pakistan’s public health sector, private sector and CBOs and healthcare providers providing services to MSM and transgender women living with and without HIV. All participants were in the age range of 40 to 55 years. Most had Bachelor’s in Medicine, and Bachelor’s in Surgery (MBBS) degree or post graduate degrees in public health, all with considerable experience in health care planning/provision and policy development. Four of the interviewees were females, one was transgender-woman, and the rest of the participants were men.

Data collection was conducted between March and August 2021 by the first author (ME) who is an experienced MD and a public health researcher trained in qualitative research methods. Key informant interviews were conducted in English or in local languages (Urdu and Sindhi). The duration of the interviews ranged from 45 to 60 min. Key informant interviews were audio-recorded and transcribed verbatim. After each interview, reflexive notes/memos were written and reviewed during analysis [34].

### Instrument development

The semi-structured interview guide was developed using the World Health Organization’s Health System Conceptual Framework also known as the Building Blocks Framework [35]. The major themes of the interview guide were: (1) Perceptions about the HPV related disease burden as a public health issue among MSM and transgender women, (2) Perceptions of introducing an integrated approach to provide comprehensive care to key populations at higher risk of HIV and HPV including tailored counselling, care and treatment, (3) Perceptions of the challenges and opportunities to implement the above, focusing on service delivery, health care financing, health workforce, medicines and commodities, health management information system and stewardship for the introduction of an HPV care model for this key population, and (4) Important issues for national and local decision-making about HPV prevention services including vaccine advocacy.

### Member checking

Key informants were emailed a set of provisional categories, subthemes and themes, and asked for feedback on relevance and accuracy, for any missing information and or any additional reflection or thoughts. This type of member-checking enabled us to assess the validity of our results [36]. Four informants provided feedback on an initial set of categories and sub-themes and themes. Generally, major changes were not suggested, since most agreed they resonated with their statements and were interpreted correctly.

### Analysis

The social-ecological conceptual framework [37] informed our data analysis as we clustered our codes and created categories and themes following the different arenas described in it, i.e., factors at the individual level, at the healthcare practice setting and providers level, at the community environment level, at the health systems and policy context level.

Qualitative content analysis was used to identify the manifest and latent content of the data [38]. The whole data analysis was deductive and iterative process. The first author led the analysis and for the purpose of data cleaning (i.e., detection and removal of all typo errors, any duplicates and or inconsistencies) reviewed the primary raw data. This was followed by an open and an in-vivo coding process. For the purpose of analysis, a focused process was used to code the data according to the framework in order to reflect the scope of our inquiry. In the next step, codes were grouped into categories, and categories into subthemes. The first and third authors ME and TSA coded one interview together followed by the team of ME with AA, AH, DA coded the 5/18 key informants’ interviews together to ensure that the information was coded similarly. To enhance the trustworthiness of the analysis, 11 remaining transcripts were hand-coded separately. The team met in regular meetings to compare the codes, resolve any coding discrepancies, and finally discuss the codebook. ME, and SS finalized the categories and themes and sub themes.

### Ethical considerations

The Human Research Ethics Committee of the Aga Khan (3612 – CHS- ERC – 15) approved this study. Participants received information describing the purpose of the study, the right to be anonymous and the investigator reviewed the informed consent form with each participant and notified them of their right to withdraw from the study at any time and that their participation is completely voluntary. Where written consent proved to be problematic due to confidentiality issue, verbal consent was accepted. We obtained permission from the respondents to tape-record the interviews on the assurance of confidentiality. The interviews were conducted either in the personal offices or via videoconferencing (zoom) calls for participants located outside of Karachi.

### Patient and public involvement

Patients and/or the public were not involved in the design, conduct or reporting or dissemination plans of the research.

## Results

### Themes and subthemes

**Fig. 1:**
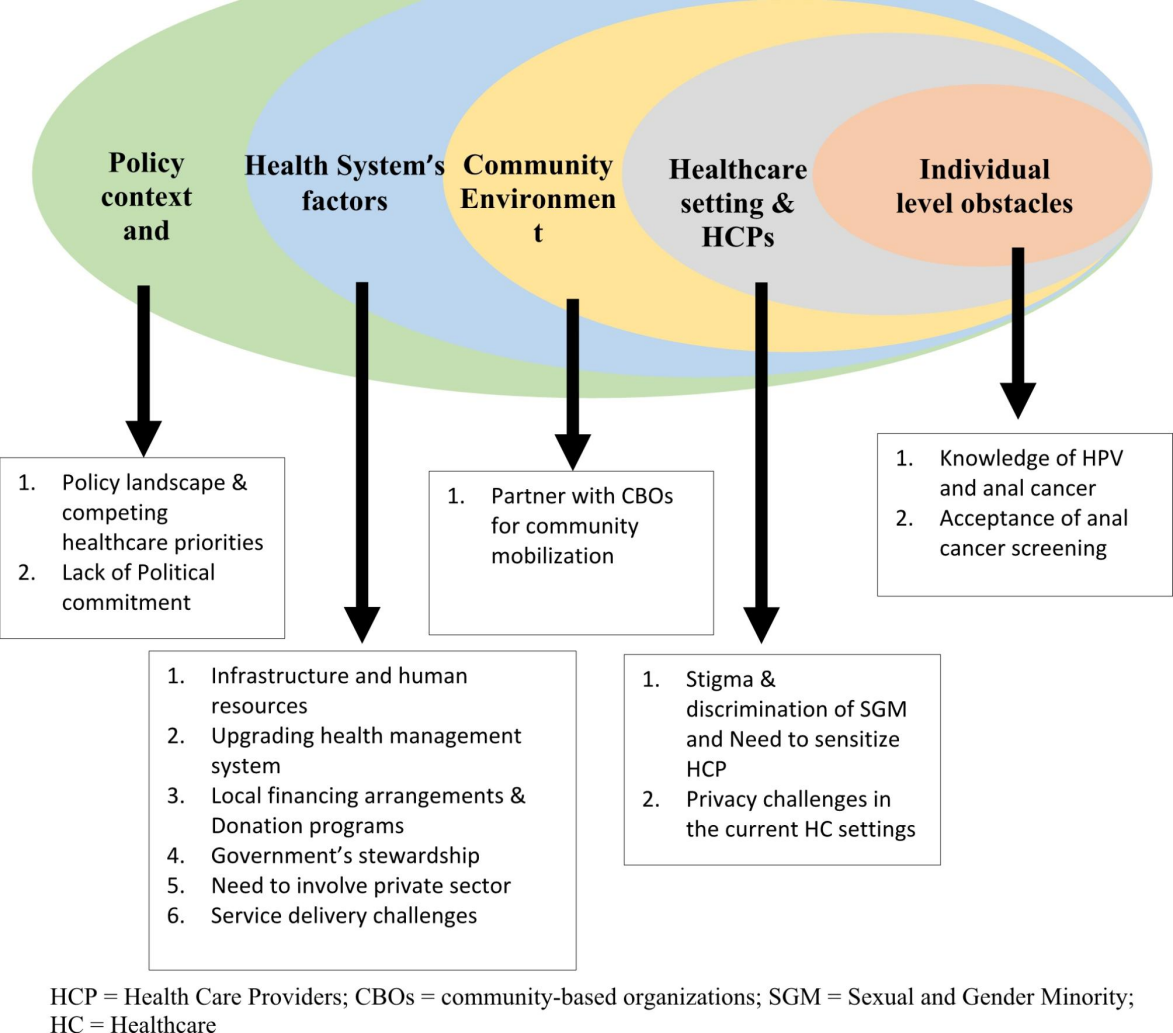
Themes and sub-themes depicting issues, and multiple factors at various levels of the socioecological framework (adopted and modified) for the integration of HPV associated anal cancer screening in existing HIV care program of Pakistan

From the data analysis, five key themes with several subthemes emerged regarding the readiness of the health system and policy environment for the integration of anal cancer screening and HPV vaccination into the existing HIV program in Pakistan. (Fig. 1).

The integrated approach to deliver HIV and HPV associated anal cancer screening services was unanimously appreciated, however, at the policy, health systems and sociocultural levels some issues and barriers were also identified. In the following section each theme and its implications will be discussed.

## Theme 1: policy context and priorities

### Policy landscape & competing healthcare priorities

Some key informants mentioned concerns regarding international donors’ influential role on health policy process and health priority settings. It was also admitted that the limited capacity of health systems to deliver basic types of services and the challenge of competing health priorities of the country could affect the operational planning of the HPV prevention for the key populations. It was argued that the competing needs i.e., emergence of communicable diseases, increasing antimicrobial resistance, prevalence of chronic diseases have put tremendous pressures on existing health systems at a time when public spending in our country in general cannot easily be increased. Moreover, participants voiced about lower public health budgets i.e., less than 3.5% of public spending on health is clearly insufficient to meet critical health needs in our country. Informants agreed that since Pakistan has not yet introduced comprehensive cervical cancer prevention strategies, it may be challenging and unfeasible to invest in screening of another target group, given limited resources.

*“Recognizing HPV as a worthy cause, in comparison to other interventions particularly for women’s and children’s health, the reduction of under-5 mortality etcetera, that currently are prioritized challenges for decision-makers, where do you put the money when you don’t have enough of it?”* (Interview # 3).

### Lack of political commitment

Most of the participants from the private sector recognized HPV as a public health issue and understood the severity of the problem, especially among key populations living with HIV. Almost all participants saw benefits of the integrated approach combining the delivery of HIV and HPV-associated anal cancer screening services. However, the government’s lack of political and leadership commitment was identified as an important barrier to succeeding with the introduction of anal cancer screening. It was mentioned that the HIV/AIDS program initiated in 1987 received limited attention until HIV positive individuals started surfacing, and the government realized that HIV was a huge problem. Informants recognized that current data on HPV infection and precancerous lesions among key populations in Pakistan point towards another potential epidemic and that the government should be proactive rather than reactive as in the case of HIV response. Regarding healthcare facilities for vulnerable populations where their specific needs could be addressed, one of the participants expressed this concern as follows:

*“The government recognizes transgender though, yet it has only established one CBO in Larkana [small city in one of the country’s provinces] for them, nothing in Karachi [the metropolitan city where the study was conducted]. They may have the capacity but lack the good will and commitment to do it” (Interview # 06)*.

## Theme 2: health systems factors

### Infrastructure and human resources

It was recognized that integrating anal cancer screening services into existing HIV programs would enable a more efficient use of health workers and infrastructure. The existing system can be helpful if appropriate guidance and a roadmap is provided. When asked about infrastructural challenges one informant responded:

*“Using existing infrastructure for implementing anal cancer screening will help in the long run-in terms of expertise, cost and sustainability. We have 28,000 healthcare facilities, out of which 45 are for HIV care and treatment. They are geographically accessible, and patients registered there could access all the services including [future] anal/cervical cancer screening. We can also include vaccination for HPV in these centers” (Interview # 12)*.

However, several challenges were also identified for the provision and implementation of HPV care services including the need for additional staff i.e., cytopathologists and more training of existing staff.

#### Additional staff required

Key informants also acknowledged that well-trained human resources for health play a central role in ensuring high quality service delivery and in enhancing patient outcomes. They identified that the relative lack of trained human resources would be an issue in any future implementation of screening, for example the fact that existing staff at the HIV clinics are not trained to perform Pap smears, and primary care facilities lack cytopathologists and technicians who can process the Pap-samples.

One participant reasoned as follows:

*“It will not be an easy task, but it could be done at the STI clinics. We would require skilled staff though; the existing staff would not be able to do it. We would need to hire more staff, more staff trained in diagnostics, and lab technicians. However, existing finances could be a challenge.” (Interview # 12)*.

#### Training of existing staff and training material

Moreover, the need for the training of existing staff through continuous professional development programs was also discussed, as visual inspection of anal lesions is a subjective test, it requires adequate training, regular supervision, and periodic reorientation of the providers, to ensure quality. The existing healthcare staff has inadequate training pertaining to this problematic medical condition. One participant stated:

*“The issue here, in my opinion, is that healthcare providers are not fully aware about this problem and the capacity of healthcare providers is limited. This can be changed through continued training and education. So, I think institutions like yours [the Aga Khan University Hospital (AKUH)] should arrange training material and training of our existing staff working in the public hospitals.” (Interview # 03)*.

### Upgrading health management information system

Participants recognized that the health management and information system (HMIS) provide the underpinnings for decision-making and needs to be more robust, in particular to allow linkages between facilities, which currently is a clear weakness.

*“The existing HMIS is weak and would not be able to monitor HPV preventive and treatment service delivery. We must work on the linkages. Information is of little value if it is not available in formats that meet the needs of multiple users, i.e., policymakers, planners, managers, health-care providers, communities, and individuals. Dissemination and communication are therefore essential attributes of the health information system” (Interview # 13)*.

Some participants said that the existing HMIS is good, but overburdened, and that this has become worse during the pandemic. It was suggested that introduction of HPV service delivery into the health management system should be done in phases, possibly preceded by a pilot study in major HIV centers. A successful pilot project could thereafter be used as a model at provincial and national level.

### Local financing arrangements & donation programs

Public sector participants explained that the provincial health department is responsible for the financing of the secondary preventive services. However, a robust budget proposal and expert support is required to make a strong case. Moreover, the informants suggested that the government should take control of HPV prevention services, as it has the capacity and resources to successfully administer vaccination and anal cancer screening program.

It was proposed that existing evidence on the benefits of HPV vaccination and anal cancer screening should be presented to the planning and development forum for funding, and cost be divided between the federal and provincial governments. One of the participants stated:

*“The provincial government has a public health wing, under which they have a development wing. They will present this [HPV infection and precancerous lesions] data to the planning and development forum of the province, and we will justify why this program needs to be introduced. If it’s too costly, then they will ask the federal government to cover some of the cost and the rest can be covered up by the provincial government.” (Interview # 08)*.

#### Donation programs

Besides the federal and provincial governments, other possible funding sources were also discussed. For example, informants mentioned that they could approach international donors, such as the World Bank and the Global Alliance for Vaccines and Immunization (GAVI) to fund this enterprise partially or totally. It was suggested that Pakistan could negotiate a long-term deal with pharmaceutical companies to make HPV vaccines more affordable and sustainable for the country.

*“External funding in our country has a major contribution to our resources to health, we can ask the World Bank to invest some amount [40%] and the remaining amount [60%] could be taken as a loan.” (Interview # 10)*.

One participant emphasized the need to search for multiple sources of funding and highlighted the importance and potential use of locally available resources such as the Islamic wealth tax [zakat], the contribution of the private sector and charity. According to this interviewee all these various resources could be useful if combined with global funds.

*“For long-term sustainability our own long-term funding solutions must be developed.” (Interview # 08)*.

### Government’s stewardship

Informants from the public sector felt that the government should take a more proactive role in the provision of STIs services to sexual and gender minority groups. However, for sustainability, it was also recognized that this must be done in close collaboration with the CBOs and all relevant actors that currently provide health care for these key populations.

*“It has to be the government. It’s not possible without the government, but it should utilize the funds properly. They have a program on communicable disease control (CDC), HIV comes under CDC and HPV can easily be integrated within HIV services” (Interview # 18)*.

However, it was also recognized that for sustainability everyone that has a role to play must be involved and provides the support that is needed.

### Need to involve private sector

Participants from the private sector, however, strongly felt that they should be given a leading role in any future HPV related program, mainly due to governmental malpractice and misappropriation of funds. Public-Private Partnership (PPP) was proposed as a possible way forward to combine the strengths of both public and private actors and to effectively reach the target population. For example, it was proposed that the public sector could oversee governance and financing activities while program implementation could be conducted by CBOs and other private institutions. Informants argued that PPP model could be effective since CBOs already provide social support & health education to vulnerable groups and are supportive of government collaboration.

*“The government needs to involve all the subgroups of the community, they all need to be on the same page, and all have to agree, because such advocacy influences implementation and policy makers.” (Interview # 16)*.

### Service delivery challenges

Although most agreed that it would make sense to combine and integrate services for HIV and anal cancer screening, some implementation challenges were also identified.

#### Inadequate referral systems

Participants had mixed views with regards to the capacity of different health facility levels to integrate and properly refer the patients in need of more advanced surgical treatment. From the public sector point of view few were optimistic that the system is ready to address the issue of HPV. However, others identified a clear need for government led referral centers for more specialized services. One participant stated that:

*“For specialized services you would need robust referral centers, which should ideally be run by the government. It would be helpful, as integration is not possible everywhere, if there are two-three referral centers where treatment is provided, that’s enough.” (Interview # 11)*.

#### Lack of proper and advanced diagnostics services

The lack of advanced diagnostic services including trained staff that can perform biopsies during High Resolution Anoscopy (HRA) for the histological assessment of the precancerous lesions was identified as a major bottleneck for service delivery.

*“If the cases are being identified at the initial cytology screening, we then don’t have the required advanced diagnostic facilities, neither the equipment nor the trained eyes. That’s our system’s shortcoming.” (Interview # 05)*.

## Theme 3: community environment

### Partner with CBOs for community mobilization

Our results indicate that public knowledge and acceptance, early community involvement and social mobilization are cornerstones in the introduction of almost any preventive intervention. It was suggested that public awareness and community engagement would facilitate both primary and secondary prevention by tackling issues like embarrassment associated with STIs and community acceptance of HPV prevention. Study participants recognized the need for a sensitization campaign as well as for empowering the gay communities.

*“We need not only to sensitize the members of the community, but also to put them in charge of the issue. The main issue is lack of empowerment. This can be changed by disseminating information and knowledge amongst them. Next, you must work towards restoring their faith in our healthcare systems.” (Interview # 11)*.

## Theme 4: practice setting and healthcare provider level barriers

### Stigma & discrimination of SGM community: Need to sensitize HCP

The key informants stated that discriminatory and stigmatizing attitudes towards sexual and gender minorities are common at all levels, i.e., family, community and societal. They also acknowledged that SGM stigma is a barrier for both the screening and treatment of HPV-associated anal cancer. One of the study participants said that religious beliefs in Pakistan undermines people’s willingness to recognize the fact that their society could be affected by such disease [sexually transmitted disease including HIV]. Participants acknowledged that the public sector health workers must be properly trained to approach the target groups with tailored services. They suggested stigma-free one-stop clinics with trained staff that can provide both screening for various diseases, as well as other basic services, or else anal cancer screening could be provided in HIV centers where at least the situation is relatively better for SGM than in other healthcare services.

To the question “What challenge the system would face while offering vaccination to the key populations as well as to the young girls?” one participant responded:

*“Cultural taboo and stigmatization, people would relate HPV to sexual activity. We would face a lot of resistance if we were to introduce it amongst girls aged 9 to 12. We could involve UNICEF to develop a communication tool to address such challenges, it must be done in way which does not hurt the sentiments of the community” (Interview # 17)*.

Moreover, during the interviews, participants discussed people’s general unawareness of how to deal with vulnerable communities, and that this situation is worsened by the fact that healthcare staff often lacks appropriate training. Participants from CBOs reported that such negative attitudes prevent optimal utilization of healthcare services, and community [SGM] either delay or avoids care due to harassment or discrimination.

Moreover, they emphasized on the need to sensitize the healthcare providers to the health needs of the MSM and transgender women.

One participant explained:

*“Physicians’ homophobia is a barrier to health care. We need to train our healthcare workers to be sensitive to the needs of key population members. Most healthcare workers are unaware of their needs and that these may differ from that of the general population.” (Interview # 11)*.

### Privacy challenges in current healthcare settings

Participants admitted that current arrangements are inadequate to ensure privacy for patients from sexual and gender minorities in the existing healthcare system. This reduces healthcare access for vulnerable sub-groups, and the lack of confidentiality undermines the quality of healthcare for many at potential risk. One of the participants stated:

*“Health facilities are rarely SGM [sexual and gender minority]-friendly, no specific space for them and experiences of discrimination inside health facilities is very common in our country.” (Interview # 11)*.

## Theme 5: individual level obstacles

### Knowledge of HPV and anal cancer

Some of the study participants explained how lack of awareness and proper knowledge of HPV among members of the SGM community would pose barriers in availing HPV screening services. High-risk groups coming to the HIV clinic must be given information about anal cancer and its link to HPV.

“*First and foremost, community members need to be educated and made aware of the HPV acquisition and its transmission mode. Initially, when HIV came, nobody knew anything, just like we spread awareness about HIV, we need to do the same for HPV and HPV-associated diseases as well.” (Interview # 16)*.

### Acceptance of HPV preventive service (anal cancer screening)

Clinicians and CBO managers highlighted the need to explore the target population’s perceptions about the acceptability of anal cancer screening and their willingness to be re-screened at regular intervals. It was suggested that if screening was to be introduced, much more information would be needed to address these knowledge gaps among key populations.

## Discussion

We have recently reported a very high prevalence of HPV infection [6] and associated anal precancerous lesions [7] among MSM and transgender women living with and without HIV in Pakistan. The current study explored potential strategies for HPV and anal cancer prevention, and early detection of potential anal cancer lesions among MSM and transgender women. We explored key informants’ views of the readiness of the existing health system and the policy environment to provide HPV-associated anal cancer screening for MSM and transgender women. To the best of our knowledge this is the first study in the L&MIC region to provide insights on this issue for this key population.

The healthcare system in Pakistan is struggling with inequity, scarcity of resources, inefficiency, lack of trained human resources, gender insensitivity and structural mismanagement [39]. Although all participants recognized the magnitude of the problem, and public health implications of the high prevalence of HPV and anal cancer precursors in the key population of interest [6, 7], they emphasized the need for government and policy makers to take the lead. Key factors related to national level leadership that can facilitate integration of HPV services within existing HIV program includes a political will to implement integration, defining integration as an explicit goal and having a vision and clearly defined strategy for implementation. Moreover, the integration of vertical programs such as HPV and HIV could be facilitated by new structural and program design features possibly spearheaded by a few larger facilities with leading senior managers. Moreover, the availability of explicit guidelines and protocols checklist for the management of HPV associated disease would facilitate the proposed integrated model.

Consistent with previous research [40–42], the interviewee acknowledged the feasibility of nesting cancer control program associated with HPV infection into healthcare in general, and HIV care in particular. Almost all categories of participants thought that setting up another disease specific or verticalized program is neither desirable nor possible. An innovative service delivery approach to reach the key population by integrating anal prevention strategies into existing HIV services would promote efficient use of common health personal and infrastructure [43]. This is the case in Zambia where integration allowed for sharing of resources and infrastructure [44].

Placing at-risk populations at the center of service delivery and integrating health services as a result of policy reforms, would ensure that the health needs of the susceptible population are comprehensively met [45]. Increasing preventive care will contribute to improve healthy life expectancy and an overall quality of life of MSM and transgender women in Pakistan. Evidence suggests that screening HIV negative MSM for anal cancer could improve quality-adjusted life expectancy at a cost comparable to other preventive interventions such as screening all women in certain age groups for cervical cancer every three years [46].

In Pakistan, HIV prevention and care services are available at multiple public and private designated centers as well as at CBOs in all large metropolitan cities. Screening for anal precancerous lesions is a simple intervention that easily could be integrated into routine care visits to HIV facilities, as suggested by others also [47]. Health care providers at HIV service facilities can adopt simple and specific algorithms for screening of anal precursors as a standard of practice [42], and is already well accepted by MSM [48]. However, the type of human resource required for screening services would depend on the structure of the program [49]. While a need for training of staff in new screening techniques was recognized, integration of HPV related services would provide a unique opportunity for providers to increase their knowledge and skills. Moreover, the stability of a trained workforce in Pakistan as in many other resource constrained countries is compromised by high-turnover and lack of consistency of contracts for health care professionals [49]. Training and utilization of community health volunteers/ workers, task shifting [50] of care (especially treatment) from referral centers to the primary levels has been successfully implemented elsewhere to address scarcity of trained medical expertise [49].

As identified by many of the study participants that there is a dire need for implementing an effective information system as an integral component of a good health system and effective disease control program, would be required to keep track of the number of HPV prevention services provided at the facility-level. Moreover, developing and collecting individual-level indicators will allow effective monitoring and measurement of both scale and quality of the integrated service delivery [49].

For introduction and sustainability of anal cancer screening program, lack of domestic finances was identified as an important limitation, hence many participants saw a need to engage with global partners for initial funding. For example, HIV programs in low and middle income countries are currently gaining substantial support from partners like the Global Fund [40]. Additional sources of extramural funding can be mobilized to increase the provision of HIV testing, treatment, and care. These investments can be extended by integrating anal cancer screening services and in turn open the doors for further funding. Pakistan is a GAVI eligible country, and WHO, UNICEF and UNAIDS are the other donors that can also be approached for funding primary prevention through vaccination for not only high-risk groups but for girls at large. In the long run funds should be made released from domestic sources such as the Ministry of Health and Finance of the government of Pakistan. International donors working with governmental, non-governmental and private organizations receive funding from separate funding streams. Through increased coordination among existing implementers, the government could optimize resources for HPV care services to increase access to anal lesions screening and preventive services.

Furthermore, as CBOs administrators identified, members of key populations are often reluctant to seek healthcare in both public or private facilities because they fear being identified, stigmatized, and ridiculed. However, the environment at current HIV care services both the public and private sectors, is welcoming to individuals of all sexual orientations. Issues of sexual minority identity disclosure may be less of a problem at HIV care services and a sense of connectedness between patient and provider, and discussions of sexual health at these services could facilitate addressing anal health. HIV care services also offer culturally competent sexual health communication and care. Furthermore, scientific evidence exists that disclosure of sexual identity or orientation is associated with acceptability of receiving preventive services [51, 52]. Strong leadership promotes a shared vision among multiple stakeholders who are committed to changing the current social landscape by openly addressing stigma and committing resources to improve the well-being of SGM.

### Strengths & limitations

Most informants of our study were heavily involved in and knowledgeable about health care service delivery, policy development and implementation in Pakistan, and provided us with ample insights. Other methodological strengths included the use of WHO’s Health Systems Framework for data collection, the socioecological model for data analysis, and the validity checks of findings with key informants.

Although we did reach saturation of information, the number of respondents was partly limited by some potential candidates declining interview due to the COVID-19 situation. However, the qualitative nature of the study does not require large sample size and consequently study findings are not meant to be generalizable. Another limitation may be selection bias given our snow-ball sampling methods starting in our existing networks. Only informants willing to share their time and expertise participated in the study. Nevertheless, we were able to recruit a diverse group of systems, policy, and other healthcare administrators. Risk of subjectivity is another potential limitation, but this was minimized by independent analysis of the data by multiple co-authors followed by joint discussion of interview contents, coding, and sub-themes in regular meetings throughout the data collection and analysis period.

## Conclusions

Guided by our conceptual framework for the analysis of data, this study indicated that effective introduction of anal cancer screening and HPV testing for the members of sexual and gender minority living with and without HIV can be influenced by multilevel factors and that political support and a context specific implementation plan for HPV screening is required that is well integrated within the existing HIV prevention program. Moreover, the government should tailor those implementation plans in a way that they are resilient to country’s known health systems fluctuations in workforce and financing. Fact of the matter is that the multi-sector collaboration between public health, academic institutions, community-based sectors would be effective in introducing HPV associated anal cancer preventive services in L&MIC in general and Pakistan in particular. To inform the development of screening guidelines, more rigorous studies need to be conducted. Education to reduce the prevailing prejudice among healthcare providers towards talking about sexuality and sexual identity is fundamental to eliminating health seeking barriers.

## Data Availability

The data that support the findings of this study are available from Global & Sexual Health Research Group of Department of Global Public Health on reasonable request and with permission of Karolinska Institutet (KI) Stockholm Sweden. Dr Muslima Ejaz can be contacted at the following email address. Muslima.ejaz@aku.edu.

## Declarations

### Patient and public involvement

Patients and/or the public were not involved in the design, conduct or reporting or dissemination plans of the research.
